# Supplementary material for: A deep learning approach reveals unexplored landscape of viral expression in cancer
Source: Nat Commun. 2023 Feb 11;14:785. doi: 10.1038/s41467-023-36336-z (PMC9922274; doi:10.1038/s41467-023-36336-z)
Supplement: Supplementary file 1 — Supplementary Information [file 41467_2023_36336_MOESM1_ESM.pdf]

Supplementary Information for: A deep learning approach reveals unexplored landscape of viral expression in cancer

Supplementary Tables

Cancer type	Virus	Unadjusted Log-rank p-value	FDR Adjusted Log-rank p-value for all virus-disease associations
CESC	High risk HPV	0.71	1
LIHC	HBV	0.042	1
LIHC	HCV	0.649	1
HNSC	High risk HPV	0.045	1
KIRC	High risk HPV	0.95	1
SKCM	HBV	0.649	1

Supplementary Table 1. Survival associations between oncoviruses and cancer types.

	sample	virus	Avg similarity	Avg coverage	Max coverage	Contig #
Assembly using model	6dd4e6c6-db0c-47be-ab00-883418a57f67	NC_003977	92.9581889	75.9222222	100	86
	5c57b82a-e94f-43e2-a281-f5b2c5d030c4	NC_003977	93.4691543	78.4571429	100	170

	852a326d-d81f-43df-96d5-256db17f13a4	NC_003977	92.8479904	92.2250804	100	297
	d34e8f7d-bdb0-41e6-9b28-d50189d68cda	NC_003977	92.7826667	92.4444444	100	9
	4c20ae1d-ad19-4424-a62b-622c4fbf4397	NC_001401	97.3660631	96.6396396	100	106
	90fca208-cdd6-4a29-85ee-f1e27fe4a536	NC_003977	94.7749688	68.4270833	100	95
	a0f39d3d-bf2f-4e4e-82c7-b6bdb45ee0d4	NC_003977	94.6758222	72.4222222	100	44
Assembly not using model	6dd4e6c6-db0c-47be-ab00-883418a57f67	NC_003977	93.390442	76.7898551	100	133
	5c57b82a-e94f-43e2-	NC_003977	93.8706037	80.262963	100	254

	a281-f5b2c5d030c4					
	852a326d-d81f-43df-96d5-256db17f13a4	NC_003977	92.5571439	91.9256595	100	403
	d34e8f7d-bdb0-41e6-9b28-d50189d68cda	NC_003977	94.1901667	82.0833333	100	12
	4c20ae1d-ad19-4424-a62b-622c4fbf4397	NC_001401	96.811156	95.7092199	100	135
	90fca208-cdd6-4a29-85ee-f1e27fe4a536	NC_003977	94.7448125	70.5234375	100	126
	a0f39d3d-bf2f-4e4e-82c7-b6bdb45ee0d4	NC_003977	94.2369286	73.875	100	55

Supplementary Table 2. Comparison of naïve assembly with and without using model scores over 10 LIHC samples

Supplementary Figures

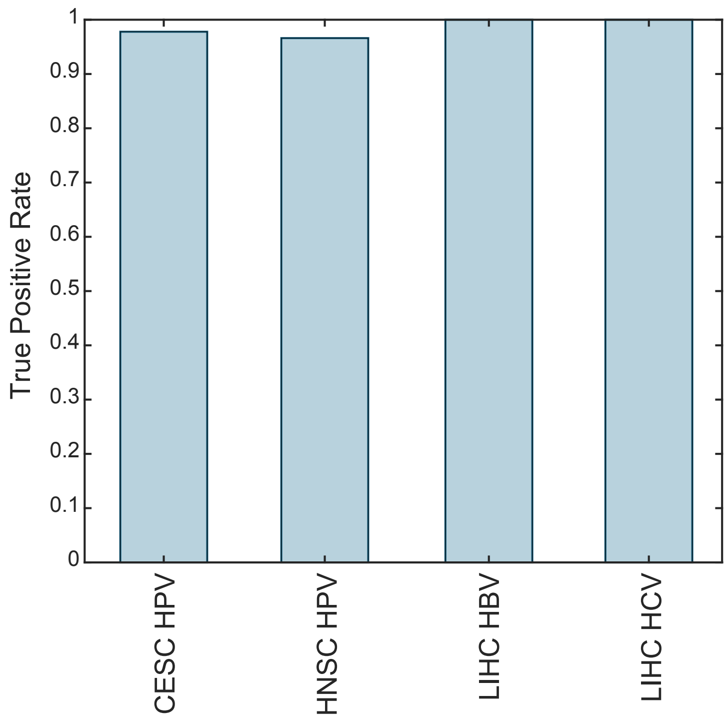

Supplementary Figure 1. The proportions of TCGA samples that are identified as virus-positive by viRNAtrap that were also verified as virus-positive through TCGA clinical information. From left to right: HR-HPV-positive in CESC, HR-HPV-positive in HNSC, HBV-positive in LIHC and HCV-positive in LIHC. HR-HPV: high-risk human papilloma virus; HBV: hepatitis B virus; HCV: hepatitis C virus.

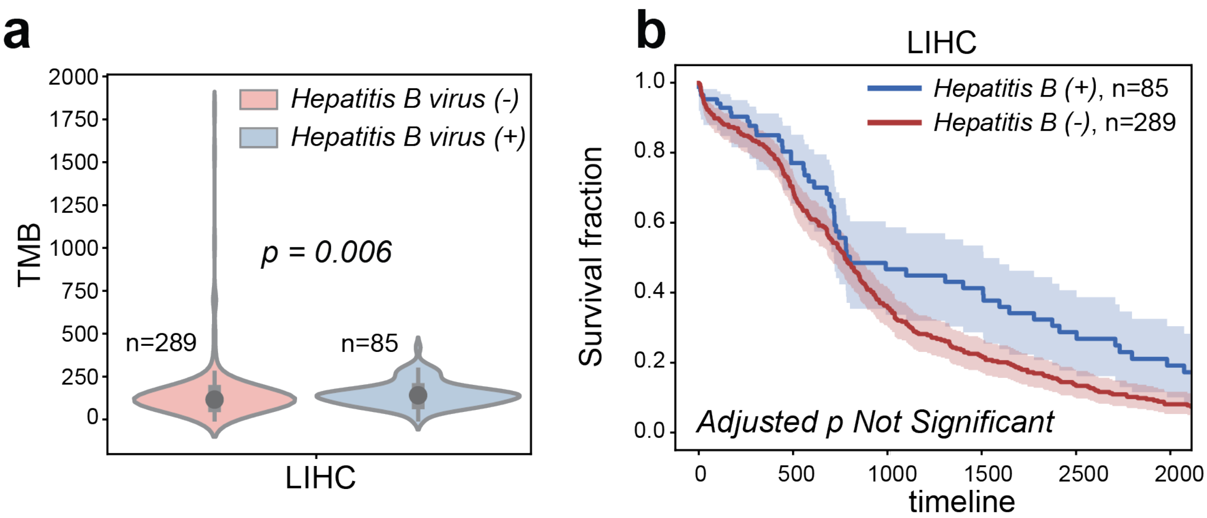

Supplementary Figure 2. Hepatitis B viruses correlates in LIHC patients. (a) Violin plots comparing the tumor mutation burden (TMB) between LIHC patients where expression of Hepatitis B viruses was detected vs those patients where it was not detected. Black dots represent the medians, and the boundaries of the violin plots refer to the maximum and minimum values, respectively. (b) Kaplan-Meier curves comparing the survival rates between LIHC patients where the expression of Hepatitis B viruses was detected (blue curve) vs those where the expression of Hepatitis B viruses was not detected (red curve). For Kaplan–Meier curves, shaded areas represent the confidence interval of survival. The FDR adjusted p-value is not significant (Supplementary Table 1).

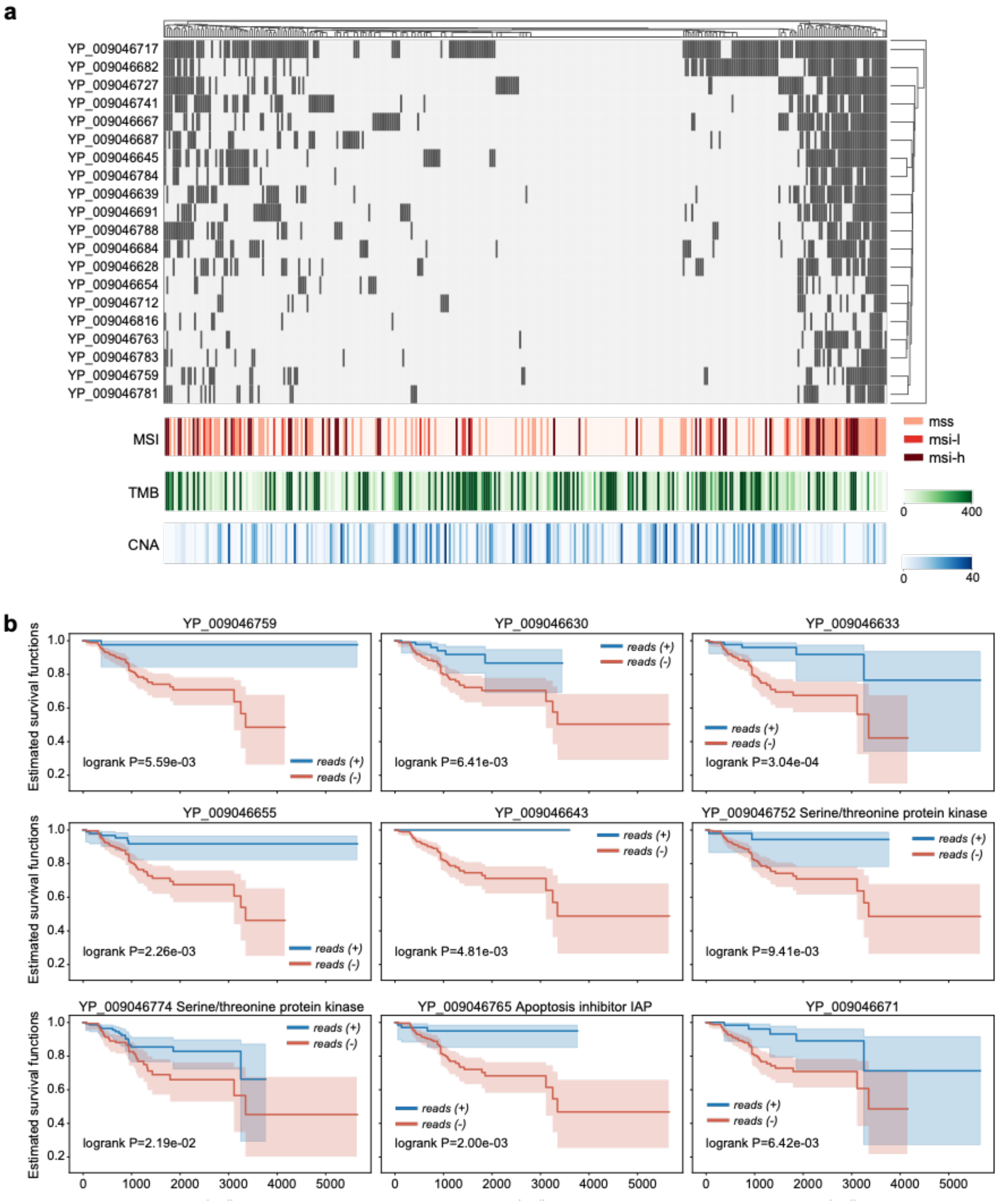

Supplementary Figure 3. IIV31 correlates in UCEC patients. (a) Heatmaps showing IIV31 proteins expressed in different tumors, microsatellite instability, chromosomal aneuploidy, and tumor mutation burden (TMB) across endometrial cancer samples. For Kaplan–Meier curves, shaded areas represent the confidence interval of survival

(b) Kaplan-Meier survival curves comparing survival based on presence (blue) or absence (red) of different IIV31 proteins in endometrial cancer samples.

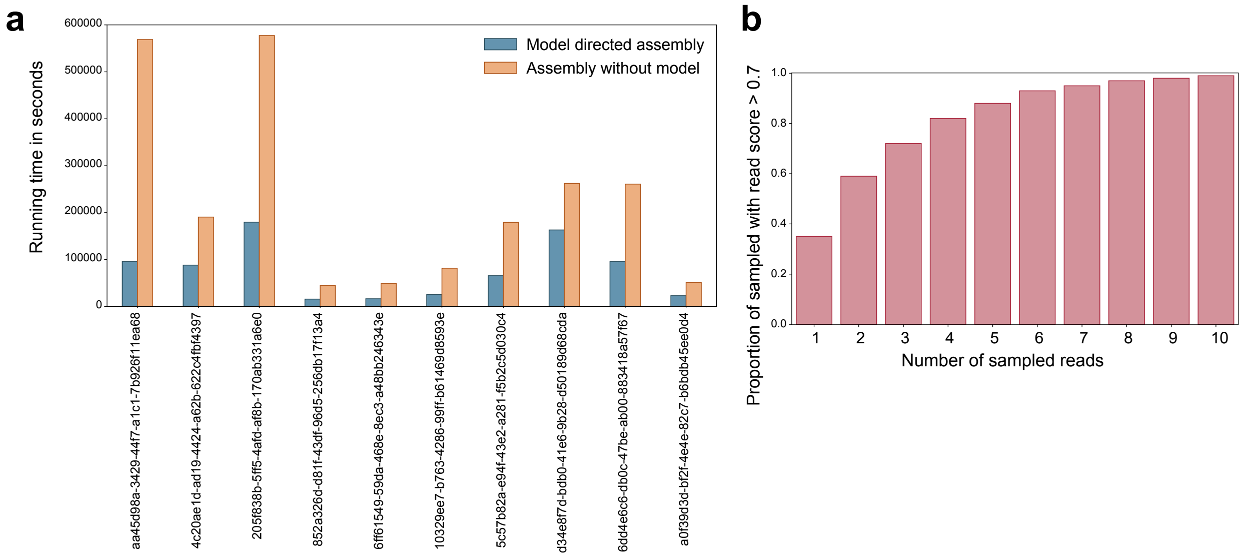

Supplementary Figure 4. viRNAtrap algorithm evaluation. (a) Running time (seconds, y-axes) comparison of naïve assembly with and without using model scores over 10 LIHC samples. The naïve assembly that is not using the model scores takes up to 6 times longer to complete. (b) Simulation analysis to evaluate the number of viral reads for identification with the viRNAtrap model score threshold of 0.7. From 10,000 randomly sampled groups of viral reads from the test dataset with different group sizes (x-axis), the proportion of groups with at least one viral read scored above 0.7 (y-axis).

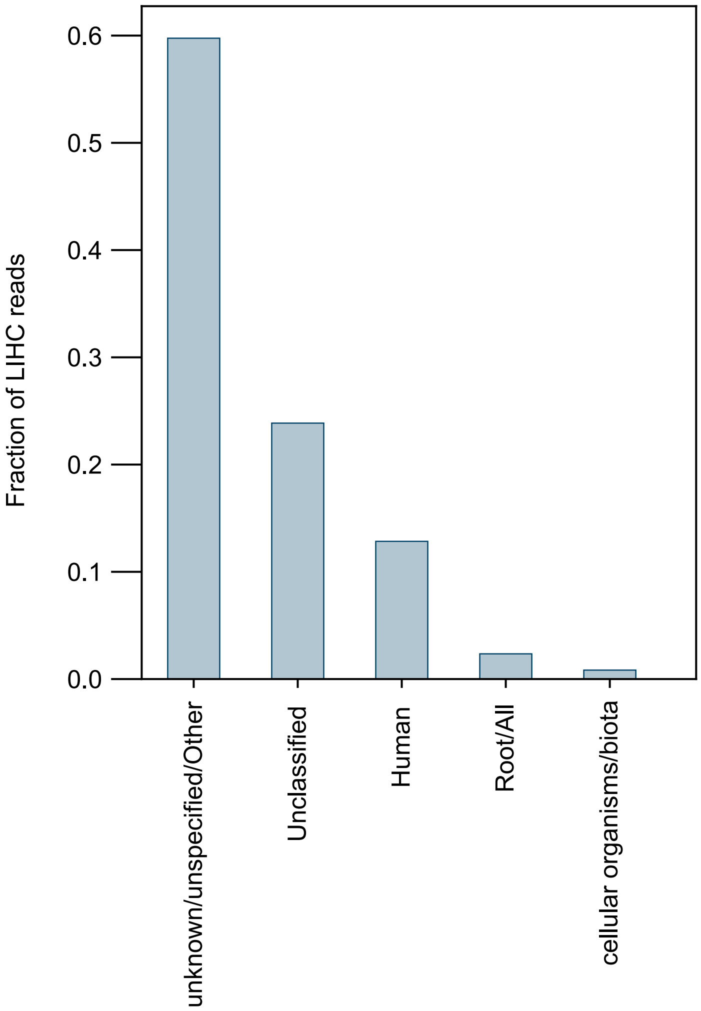

Supplementary Figure 5. Kraken2 evaluation for LIHC RNAseq reads. Barplot showing the classification of LIHC reads (which are 48bp), that were unmapped to human and the Phix phage by Kraken2.

Supplementary methods

<u>Reverse-transcriptase qPCR (RT-qPCR)</u>

RNA was extracted using TRIzol reagent (Invitrogen, cat. no. 15596026). Extracted RNA was used for reverse-transcriptase PCR using a High-capacity cDNA reverse transcription kit (Thermo Fisher, cat. no. 4368814). Quantitative PCR was performed using a QuantStudio 3 real-time PCR system. GAPDH was used as an internal control.

The fold change was calculated using the $2^{-\Delta\Delta Ct}$ method. The primers used for reverse-transcriptase qPCR are:

GAPDH forward, GTCTCCTCTGACTTCAACAGCG and reverse, ACCACCCTGTTGCTGTAGTAGCCAA.

COV318 contig1 forward, TTGCGATGCGTACTCAGACT and reverse, 5'-CTCTTTTTGGTCAGCAGCGG-3'.

The primers are designed based on the template:

>contig1[[0.9004159]] terminase

TTCCGATCTCCTTGGCCGCATATTGCGATGCGTACTCAGACTACATCAAATGCACA
AAAATCATTCAAGAAGAAGGTTTGATGGTTGAGTACACCAACAAGGCAGCTGAAAC
CAATAAAGTCGCTCATCCGCTGCTGACCAAAAAGAGACAACTGTTC

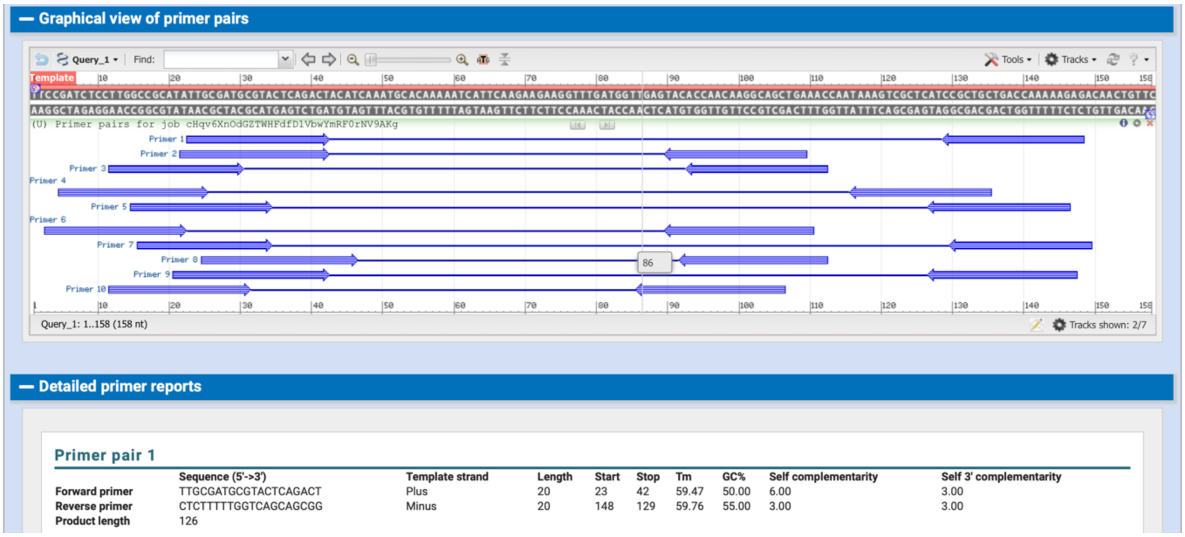

Training existing methods for virus identification

1. *DeepViFi.* We trained DeepViFi as instructed in the method's github repository, https://github.com/UCRajkumar/DeepViFi. A transformer was trained using the parameters defined in the configuration file, with embedding dimension of 128, 16 heads, 8 layers, the feed forward dimension set to 256 and the batch size set to 256. The

generated embedding by the transformer for each sequence read was used to train a random forest classifier using the transformer representation (through sklearn.ensemble), with 500 trees as recommended by DeepViFi.

2. *DeepVirFinder*. We followed the instructions of DeepVirFinder github repository: https://github.com/jessieren/DeepVirFinder to train a model and evaluate it using our data. Even though DeepVirFinder was developed to take various input sizes (300bps, 500bps and 1000bps), there is an option to choose input size less than 300bps, which we used by setting the input size to 48. We used the parameters as defined by the authors to train the model as following: dropout convolutional neural network (CNN) of 0.1, dropout pool of 0.1, learning rate of 0.001 and number of filters of 500, of which each of length of 10.

3. *ViraMiner*. The ViraMiner model was trained as end-to-end CNN model as instructed in its github repository, https://github.com/NeuroCSUT/ViraMiner. The model was trained with filter size 8, dropout of 0.1, learning rate of 0.001 and layer_size of 1000. Even though the input sequence length in the original method was defined to be 300bps, we modified the code (specifically, we modified helper_with_n.py line 73 from 300 to 48) to accept input sequences of size 48bps.

4. *Off-the-shelf seq2seq*. We trained off-the-shelf seq2seq model using Keras (with LSTM components) on our data by configuring the model to take 48bp input sequences and the embedding size was defined to be of size 64 while the learning rate was set to 0.001. Then, to accommodate to DeepViFi, which also compared their representation against off-the-shelf seq2seq model, the seq2seq representation of viral sequences was given as input to a random forest classifier (using sklearn.ensemble) with the same parameter defined, the number of trees, to be 500.
