# Supplementary material for: A deep learning approach reveals unexplored landscape of viral expression in cancer
Source: Nat Commun. 2023 Feb 11;14:785. doi: 10.1038/s41467-023-36336-z (PMC9922274; doi:10.1038/s41467-023-36336-z)
Supplement: Supplementary file 3 — Description of Additional Supplementary Files [file 41467_2023_36336_MOESM3_ESM.docx]

Supplementary Datasets

**Supplementary Data 1.** Average model scores assigned to different human viruses plotted in Figure 1d. Model scores were averaged across all 48 bp segments for each virus, using 2bp window size.

**Supplementary Data 2.** RefSeq viruses identified in 7272 TCGA samples from 14 cancer types, together with tumor mutation burden (TMB), chromosome level copy number alterations (CNA) overall survival time and death (0=survival, 1=death) for each TCGA sample considered in this study.

**Supplementary Data 3.** Complete information for viruses identified in 7272 TCGA samples from 14 cancer types providing the number and lengths of contigs as well as Blast parameters.

**Supplementary Data 4.** Human endogenous viruses identified in 7272 TCGA samples from 14 cancer types. The ERV identifier can be mapped via Supplementary Table 5 to the hg19 genomic interval that contains the ERV.

**Supplementary Data 5.** Intact retroviral genes, chromosomal location (hg19 assembly) and human gene and distance (measured as minimum between the number of bp from the start and end of each gene, where intronic HERVs are not distinguished) of each HERV to the nearest gene identified in TCGA tumor samples. The distance from the nearest SNP (dist_from_SNP) and the phenotype associated with the nearest SNP (SNP distance) are provided, and -1 values are assigned if no disease associated SNP was found located near a HERV.

**Supplementary Data 6.** Associations between HERV presence and tumor mutation burden or chromosomal aneuploidy across the 14 cancer types from TCGA. The values correspond to one sided Wilcoxon rank-sum p-values. TMB_greater and TCNA_greater test whether the TMB or CNA is greater in the presence of each HERV, and TMB_less and TCNA_less test whether the TMB or CNA is lower in the presence of each HERV.

**Supplementary Data 7.** Hyper-geometric enrichment p-values evaluating enrichment between somatic mutations in 10 frequently mutated cancer driver genes, and the expression of 36 HERVs that were found frequently expressed in cancer tissues.

**Supplementary Data 8.** Somatic mutations in frequently mutated cancer driver genes for cancer types in which HERV expression was associated with poor survival, and HERVs identified in TCGA samples within these cancer types.

**Supplementary Data 9.** Divergent unexpected viruses found expressed in 7272 samples from 14 cancer types from TCGA used throughout this study.

**Supplementary Data 10.** The IIV31 proteins identified in endometrial cancer (UCEC) samples with the tumor mutation burden and chromosomal aneuploidy scores. -1 values are assigned to samples with RNA sequencing data that did not have mutation or copy number information to evaluate the TMB or CNA.

**Supplementary Data 11.** Identified contaminants accessions that were excluded from analysis.

**Supplementary Data 12.** HERV and cancer type survival analysis, with FDR corrected log-rank p-values that was applied per cancer type and globally.
