# Supplementary material for: A deep learning approach reveals unexplored landscape of viral expression in cancer
Source: Nat Commun. 2023 Feb 11;14:785. doi: 10.1038/s41467-023-36336-z (PMC9922274; doi:10.1038/s41467-023-36336-z)
Supplement: Supplementary file 16 — Reporting Summary [file 41467_2023_36336_MOESM16_ESM.pdf]

nature portfolio

Corresponding author(s): Noam Auslander

Last updated by author(s): 01/19/2023

Reporting Summary

Nature Portfolio wishes to improve the reproducibility of the work that we publish. This form provides structure for consistency and transparency in reporting. For further information on Nature Portfolio policies, see our Editorial Policies and the Editorial Policy Checklist.

Statistics

For all statistical analyses, confirm that the following items are present in the figure legend, table legend, main text, or Methods section.

n/a	Confirmed	
☐	☒	The exact sample size (*n*) for each experimental group/condition, given as a discrete number and unit of measurement
☐	☒	A statement on whether measurements were taken from distinct samples or whether the same sample was measured repeatedly
☐	☒	The statistical test(s) used AND whether they are one- or two-sided<br>*Only common tests should be described solely by name; describe more complex techniques in the Methods section.*
☐	☒	A description of all covariates tested
☐	☒	A description of any assumptions or corrections, such as tests of normality and adjustment for multiple comparisons
☐	☒	A full description of the statistical parameters including central tendency (e.g. means) or other basic estimates (e.g. regression coefficient) AND variation (e.g. standard deviation) or associated estimates of uncertainty (e.g. confidence intervals)
☐	☒	For null hypothesis testing, the test statistic (e.g. *F*, *t*, *r*) with confidence intervals, effect sizes, degrees of freedom and *P* value noted<br>*Give P values as exact values whenever suitable.*
☒	☐	For Bayesian analysis, information on the choice of priors and Markov chain Monte Carlo settings
☐	☒	For hierarchical and complex designs, identification of the appropriate level for tests and full reporting of outcomes
☐	☒	Estimates of effect sizes (e.g. Cohen's *d*, Pearson's *r*), indicating how they were calculated

Our web collection on statistics for biologists contains articles on many of the points above.

Software and code

Policy information about availability of computer code

Data collection	We downloaded RNA-sequencing data from Genomic Data Commons (GDC; https://portal.gdc.cancer.gov/) as BAM files. High quality reads were selected and mapped with Bowtie2 against hg19 (1000 Genomes version) and PhiX phage (NC_001422), and only the unmapped reads were kept. Then, we merged the paired end reads and converted them to fastq files, which were used as input to for the viRNAtrap framework, to yield predicted viral contigs.
Data analysis	Quality standards for virus identification<br>For all viruses, blastn was applied with E-value cutoff of 0.01 and any sequences with a match to contaminant accessions (that were associated with vector contamination) were filtered out.<br>a. Reference viruses. For every sample, contigs mapped to each accession were extracted. Identified accessions with maximum qcov across contigs more that 90%, average qcov more than 50%, and average similarity more than 90% were considered. Accessions with maximal contig length under 100bp were manually inspected and verified against nr.<br>b. Human endogenous viruses. For every sample, contigs mapped to each HERV were extracted. HERVs with contigs longer that 200bp, and with average qcov and similarity more than 95% were considered.<br>c. Divergent viruses. For every sample, contigs mapped to each accession were extracted. Viruses already identified through the reference database were removed. Identified accessions with maximal contig length more than 300bp and qcov more than 40%, or with maximal contig length more than 100bp and qcov more than 75% and average similarity more than 75% were considered for manual inspection.<br>All instances of divergent viruses identified in TCGA samples were verified using blastn against nr, to support that the virus strain is indeed the best match to a viral contig generated by viRNAtrap. We reason that non-reference viruses (divergent viruses and viruses of non-human hosts) that were identified and verified in more than one sample were less likely to be contaminant or isolated events, whereas sample with fewer reads from such viruses may be filtered due to the strict filtering. We therefore additionally searched using the STAR aligner89 across tumor types where these viruses were identified through viRNAtrap (Supplementary Dataset 3). The following accessions were additionally searched

using STAR to increase sample coverage (as these were the most interesting divergent strains found across multiple samples): Bermuda grass latent virus (NC_032405), Armadillidium vulgare iridescent virus IIV31 (NC_024451), Geobacillus virus (NC_009552) and the Human lung-associated vientovirus (NC_055523)

Filtering contaminants
To filter vector contaminants, we applied VeScreen to the assembled contigs that have been mapped to viruses through our databases, where virus accessions associated with vector contaminants were entirely removed from the search (Supplementary Dataset 11).
In addition, we examined the application of software such as Kraken291 to the RNAseq reads for filtering reads that are not likely of viral origin, by applying Kraken2 to reads of LIHC samples. However, we found that 99% of the reads would not be filtered using this approach (Supplementary Figure 5), likely due to the short reads (48bp) for which Kraken has not been designed or evaluated, as longer sequences are known to be more accurately mapped92.

Genomic correlates of viral expression
We correlated viral expression with genomic markers across TCGA samples. Chromosomal aneuploidy levels for TCGA samples were extracted from93 and the total number of chromosome-arm-level alterations was used. The tumor mutation burden was defined to be the total number of somatic mutations in each sample, downloaded from the Xena browser94 (https://xenabrowser.net). CIBERSORT software was applied to TCGA samples using the default set of 22 immune-cell signatures.

Statistical methods
Survival analysis, including Kaplan Meier curves plots, log rank test p-values were obtained using the Python lifelines package (v0.26.4). P-values comparing TMB and aneuploidy between two groups correspond were computed with two-sided Wilcoxon rank-sum tests. Heatmap clustograms were generated through seaborn clustermap.
Viruses with significant log-rank p-values are reported as significantly associated with survival.
None of the reference viruses were significantly associated with survival after FDR correction (Supplementary Table 1), however, we report in Figure 2 the association between HR-HPV with unadjusted p-value because it is confirmatory of a known association between HR-HPV and HNSC survival.
For HERV, our exploratory data analysis uncovered some significant associations with the complete hypothesis testing. We present in the main text selected associations with at least 5 cases in each group. Nevertheless, FDR correction was applied within each cancer type for all HERV associations, and we additionally applied a global FDR correction for all comparisons across cancer types, yielding some significant associations with less than 5 positive cases. The complete significant associations between survival and viral presence are reported in the Supplementary Data 12.

Code Availability
The scripts for pre and post processing and the viRNAtrap package are available through GitHub: https://github.com/AuslanderLab/virnatrap and Zenodo under accession code: https://doi.org/10.5281/zenodo.7548375.

For manuscripts utilizing custom algorithms or software that are central to the research but not yet described in published literature, software must be made available to editors and reviewers. We strongly encourage code deposition in a community repository (e.g. GitHub). See the Nature Portfolio guidelines for submitting code & software for further information.

Data

Policy information about availability of data

All manuscripts must include a data availability statement. This statement should provide the following information, where applicable:

- Accession codes, unique identifiers, or web links for publicly available datasets
- A description of any restrictions on data availability
- For clinical datasets or third party data, please ensure that the statement adheres to our policy

Data Availability
The complete training and test data as well as viral databases generated in this study have been deposited in the Zenodo database under accession code https://doi.org/10.5281/zenodo.7548375. The results shown here are in whole or part based upon data generated by the TCGA Research Network: https://www.cancer.gov/tcga. The raw FASTQ RNA sequencing data are protected and are not publicly available due to data privacy laws, but are available under restricted access as data can be unique to an individual. Access can be obtained from the Genome Data Commons (GDC) after receiving permission via dbGaP, following the steps described in: https://www.ncbi.nlm.nih.gov/projects/gap/cgi-bin/study.cgi?study_id=phs000178.v11.p8. The processed data including viruses identified and respective statistics are available as supplementary Data 3. The complete data generated in this study are provided in the Supplementary Information/Source Data file. Source data are provided with this paper.

Viral databases
Viral contigs yielded by the assembly component were used as inputs to blastn (version 2.12.0+). Three databases were used to search for viruses (with E-value threshold of 0.01):
(1) RefSeq reference human viruses, downloaded from the National Center for Biotechnology Information (NCBI) 77, to which we added human papillomaviruses strains that are not in RefSeq from PAVE (https://pave.niaid.nih.gov). Reference viruses were searched using blastn, with default parameters except for a word size of 15 (lower than the default of 28), which was chosen to allow identification from short contigs.
(2) more divergent viruses obtained from RVDB (https://hive.biochemistry.gwu.edu/rvdb/) which was then filtered to remove non-viral elements, endogenous viruses, and accessions that were consistently not verified using blastn against the nonredundant (nr) blast nucleotide database.
(3) Human endogenous viruses. We curated a database of potentially functional HERVs through evaluation of viral protein completeness (in contrast to a previous study that evaluated HERV expression in distinct RNAseq datasets). The initial genomic locations of reported HERV elements were downloaded from the HERVd HERV annotation database (https://herv.img.cas.cz). The nucleotide sequences in hg19 for each reported HERV were extracted using twoBitToFa84. We then applied blastx blastn (version 2.12.0+) against NR with E-value cutoff of 1E-4, as well as a profile search85 against collected POL proteins, where the profile was obtained by collecting POL genes annotated in GenBank in lentiviruses (as of September 2016) and aligning their amino acid sequences using MAFFT86. Sequences with at least one identified retroviral protein motif of: POL/RT, GAG or ENV were extracted, yielding 3,044 HERVs that were considered for search in TCGA samples

(Supplementary Dataset 5). Importantly, high mutation rate of HERV prohibits most HERV sequences from aligning to the human genome in pre-processing, however, in rare cases, HERV regions that are conserved would not be identified by this approach.

Human research participants

Policy information about studies involving human research participants and Sex and Gender in Research.

Reporting on sex and gender	Not Applicable
Population characteristics	Not Applicable
Recruitment	Not Applicable
Ethics oversight	Not Applicable

Note that full information on the approval of the study protocol must also be provided in the manuscript.

Field-specific reporting

Please select the one below that is the best fit for your research. If you are not sure, read the appropriate sections before making your selection.

- [x] Life sciences
- [ ] Behavioural & social sciences
- [ ] Ecological, evolutionary & environmental sciences

For a reference copy of the document with all sections, see nature.com/documents/nr-reporting-summary-flat.pdf

Life sciences study design

All studies must disclose on these points even when the disclosure is negative.

Sample size	All samples from each study were included
Data exclusions	No data was excluded
Replication	Not applicable, because the new findings reported are through existing data that has already been generated, and are verified by multiple samples. Therefore, results they can be repeated and fully replicated using the code provided.
Randomization	Segmented human and viral sequences were randomly split (where all segments of each transcript were considered together) into balanced train, validation, and test sets (n= 8,000,000, 800,000, and 2,558,044, respectively).
Blinding	The test dataset was left out and blinded during model training to ensure selection of a model with strong performance on unseen data.

Reporting for specific materials, systems and methods

We require information from authors about some types of materials, experimental systems and methods used in many studies. Here, indicate whether each material, system or method listed is relevant to your study. If you are not sure if a list item applies to your research, read the appropriate section before selecting a response.

Materials & experimental systems

n/a	Involved in the study	
x		Antibodies
	x	Eukaryotic cell lines
x		Palaeontology and archaeology
x		Animals and other organisms
x		Clinical data
x		Dual use research of concern

Methods

n/a	Involved in the study	
x		ChIP-seq
x		Flow cytometry
x		MRI-based neuroimaging

Eukaryotic cell lines

Policy information about cell lines and Sex and Gender in Research

Cell line source(s)	OVISE cell line was purchased from JCRB; COV318 cell line was purchased from Sigma.
Authentication	Cell lines were re-authenticated by The Wistar Institute's Genomics Facility using short tandem repeat profiling using AmpFLSTR Identifiler PCR Amplification kit (Life Technologies).
Mycoplasma contamination	Regular Mycoplasma testing was performed using LookOut Mycoplasma PCR detection (Sigma). All cell lines applied in this study were tested negative for Mycoplasma.
Commonly misidentified lines (See ICLAC register)	No commonly misidentified cell lines used.
